# Milk Production and Quality of Lactating Yak Fed Oat Silage Prepared with a Low-Temperature-Tolerant Lactic Acid Bacteria Inoculant

**DOI:** 10.3390/foods10102437

**Published:** 2021-10-14

**Authors:** Mingming Zhu, Rongqing Xie, Liangyin Chen, Minghong You, Wenlong Gou, Chao Chen, Ping Li, Yimin Cai

**Affiliations:** 1 College of Animal Science, Guizhou University, Guiyang 550025, China; zmm137227531@163.com (M.Z.); lychen2011430@sina.com (L.C.); gzgyxgc3855218@163.com (C.C.); 2 Sichuan Academy of Grassland Sciences, Chengdu 611731, China; q767685757@163.com (R.X.); ymhturf@163.com (M.Y.); gwl1975117@163.com (W.G.); 3 Japan International Research Center for Agricultural Science (JIRCAS), Tsukuba 305-8686, Japan

**Keywords:** silage, lactic acid bacteria, yak milk, rumen fermentation, fat acid

## Abstract

This study aimed to investigate the effect of oat silage treated with a low-temperature-tolerant lactic acid bacteria (LAB) inoculant on milk yield and the quality of lactating yaks. Oat silages were prepared in big round bales, treated without (control) or with a low-temperature-tolerant LAB inoculant (a mixture of *Lactobacillus plantarum* BP18, *Pediococcus pentosaceus* HS1 and *Lactobacillus buchneri* LP22; the application rate of 10^5^ cfu/g on a fresh matter basis). Eighteen lactating yaks were divided into nine pairs with a similar milk yield. Each pair of yaks was randomly allocated to the control or LAB-inoculated silage treatment. The inoculated silage increased the dry matter intake and the total volatile fatty acid (mainly acetate, propionate and butyrate) in rumen fluid compared with the control. The inoculated silage also enhanced the yield of yak milk with high contents of total N, fat and lactose. In addition, high levels of essential amino acids (Thr, Leu and Phe), polyunsaturated fatty acids and low saturated fatty acids were observed in milk when lactating yaks were fed with the inoculated silage. Therefore, inoculation with a low-temperature-tolerant LAB during ensiling could promote the milk yield of lactating yaks by enhancing dry matter intake and ruminal fermentation.

## 1. Introduction

Yaks (*Bos grunniens*) are the only bovine with adaptability to the extremely harsh environment (low humidity, low temperature, low oxygen, gale and high UV radiation) of the Qinghai–Tibet Plateau at an altitude of 2000–5000 m above sea level [1]. In 2003, the global total population of yaks was estimated at 14.2 million, and about 93% of yaks were distributed in China [2]. Due to the long cold season with heavy snow, forage shortage induced a dramatic body weight loss and mortality of yaks, which caused a reduction in milk, meat, hair and cheese for the people living on the Qinghai–Tibet Plateau [3]. Yak milk is more nutritive than dairy cow milk in nutrient composition, especially for essential amino acids [4] and fat [5]. However, the milk yield and composition are dependent on feed resources [1]. Therefore, how to preserve and utilize local forage at the growing season for producing high-quality yak milk in winter and early spring is an issue of concern on the Qinghai–Tibetan Plateau.

Silage is an important source of ruminant feed in the world. Bernardes et al. [6] reviewed that the unique challenges of producing silages in cold regions lay in the fast-decreasing ambient temperature after a short growing season of forage. As a result, silage fermentation is incomplete with a poor quality, especially on the Qinghai–Tibetan Plateau [7]. The selection and application of lactic acid bacteria (LAB) strains in a low-temperature environment could promote the fermentation process and help preserve nutrients in silage. Recently, some researchers have focused on the potential of low-temperature-tolerant LAB strains during ensiling [8,9,10]. Several LAB strains from naturally fermented silage cultured at our laboratory showed a good ensiling performance on the Qinghai–Tibetan Plateau [7].

Silage inoculated with *Lactobacillus buchneri* and other heterofermentative LAB showed no detrimental effects on the intake and milk yield of dairy cows [11]. However, homofermentative LAB not only promoted silage fermentation, but also improved animal performance [12]. Oliveira et al. [13] conducted a meta-analysis of 31 lactating dairy cows, finding that homofermentative LAB increased contents of milk protein and fat, with a low interactive effect from forage type, LAB species or milk yield. Some studies attributed the improvement in milk yields and quality to homofermentative LAB-inoculated silages, because LAB inhibited detrimental microbes and toxin production [14], and interacted with rumen microbes and alteration of rumen fermentation [15]. However, the above-mentioned positive effects were only found in normal LAB strains or inoculants at temperate regions. In fact, there is still little information on the effect of silage treated with a low-temperature-tolerant LAB on animal performance.

Therefore, an experiment was designed to evaluate the effect of the low-temperature-tolerant LAB inoculant on ruminal fermentation and milk composition obtained from yaks raised on the Qinghai–Tibetan Plateau.

## 2. Materials and Methods

This study was conducted at the Hongyuan Experimental Base (N31°51′–33°33′, E101°51′–103°22′, altitude of approximately 3500 m above sea level) of Sichuan Academy of Grassland Sciences, P. R. China.

### 2.1. Silage Production

Oats were harvested in September by mower conditioners (488, New Holland Agriculture, USA), wilted to dry matter (DM) with approximately 280 g/kg of fresh weight (FW) under field conditions and divided randomly into two parts: one for no inoculation (control), and the other for treatment with LAB inoculant with the application rate of 10^5^ cfu/g on a fresh matter (FM) basis. The LAB inoculant was a mixture (2:2:1) of *Lactobacillus plantarum* strain BP 18, *Pediococcus* *pentosaceus* strain HS1 and *Lactobacillus buchneri* strain LP22, which were diluted with water, sprayed on chopped forages and mixed homogenously. The characteristics of three species in the LAB inoculant had been described in our previous study [7]. The bale silages (1.22 m × 1.25 m ø; density, about 168.9 kg DM/m^3^) were produced by BR6090 Combi from New Holland Agriculture, and wrapped with six layers of stretch film. All bales were stored at the ambient temperature and sampled after 1 year of ensiling. The chemical and microbial composition of silages was showed in Table 1.

### 2.2. Animals and Feeds

The Animal Care and Use Committee of College of Animal Science, Guizhou University, approved all procedures involving the animals. Eighteen lactating Maiwa yaks were divided into nine pairs with a similar milk yield. Each pair of yaks was randomly allocated to the control or LAB-inoculated silage treatment. Before experiment, the feeds for yaks were described by Chen et al. [16], and characterized by CP of 22.87% DM, ether extract (EE) of 14.33% DM, neutral detergent fiber (aNDF) of 10.43% DM, acid detergent fiber (ADF) of 6.99% DM, calcium of 2.45% DM, total phosphorus of 0.38% DM and NaCl of 0.62% DM. The experimental period consisted of an adaption period of 25 days and a measurement period of 5 days. Three kilograms of mixture (CP of 18.59%, crude fat of 2.73% and 18.4 MJ ME/kg) was fed to all yaks after milking. The silages were fed to each yak twice daily at 10:00 and 18:30, respectively. Residual silage in the morning was removed and weighted. The amount of silage fed to each yak was adjusted every day to ensure supply of silage was not limited. Yaks were kept in a paddock with no access to any other feeds. They could drink water at any time. Dry matter intake was determined on a daily basis. Yaks were moved and restrained in a smaller pen, and milked manually at 09:30 in the morning of experiment period. Prior to milking, the yak teats were washed with warm sterile water and dried with paper towels. The milk samples of each yak were immediately placed in sterile containers, transported via the car refrigerator at 4 °C and stored in a freezer at −20 °C. Milk samples from each yak during experiment were mixed to form a composite milk sample for analysis of chemical composition (100 mL sample).

### 2.3. Chemical Analysis of Rumen Fluids

The rumen contents were collected from each yak with an oral stomach tube [17]. In brief, the first 100 mL of rumen fluid were discarded to avoid reticulum fluid or salivary-contaminated fluid or body surface bacteria, and the subsequent 50 mL of rumen fluid were collected from each animal at the end of the experiment, and immediately measured by a pH meter (PHSJ-4F, Shanghai INESA Scientific Instrument Co., Ltd., Shanghai, China). The rumen fluid samples were thoroughly filtered with four layers of cheesecloth for analysis of volatile fatty acids (VFA) using high performance liquid chromatography [18]. The ammonia-N of rumen fluid was also measured by the method of Broderick and Kang [19].

### 2.4. Chemical Analysis of Milk

For milk samples, total nitrogen (TN) and non-protein nitrogen (NPN) were chemically analyzed by the Kjeldahl method [20]. Noncasein nitrogen (NCN) was tested according to describe by Wehr et al. [21]. Whey protein nitrogen (WPN) was calculated based on the formula of WPN = TN−NPN−NCN. Beta-lactoglobulin and alfa-lactalbumin were measured by ELISA kits (ml027529 and ml036565, respectively) according to the instructions of the manufacturer (Shanghai Enzyme Link Biotechnology Co., Ltd., Shanghai, China). Lactose content of milk was measured using the Lane-Eynon method [22].

According to the description of Liu et al. [4], 1 mL of each of the milk samples were hydrolyzed with 6.0 M HCl in vacuum-sealed tubes at 110 °C for 22 h. After hydrolysis, the samples were filtered through 0.45 m and then through 0.22 m nylon syringe filters placed in micro-centrifuge tubes (150-0045, Thermo Fisher Scientific, Waltham, MA, USA). The content of amino acids was determined using an Automatic Amino Acid Analyzer (L-8900, Hitachi, Tokyo, Japan).

Total fat acid (FA) was extracted from milk samples according to the Röse-Gottlieb method [20], which was modified by Liu et al. [4]. FA methyl esters were prepared by base-catalyzed trans-esterification according to the International Dairy Federation standard procedure [23]. FA methyl esters were determined by Sichuan Academy of Agricultural Sciences, Chengdu, China, for quantifying the FA composition of each sample using a gas chromatograph (GC2010 plus, Shimadzu Corp., Kyoto, Japan), as described by Liu et al. [4].

### 2.5. Statistical Analysis

A one-way ANOVA analysis was conducted using IBM SPSS Statistics 25.0 (SPSS, Inc., Chicago, IL, USA). The differences between means were assessed by Duncan’s multiple range method. The effect was considered significant when the probability was less than 0.05. The results were presented as mean ± S.E.M.

## 3. Results and Discussion

### 3.1. Intake and Ruminal Fermentation

As shown in Table 2, inoculation at a low temperature tended to increase DM intake of the oat silage of yaks. Monteiro et al. [24] reported that the DM intake was promoted by good fermentation and subsequently high nutrient components of LAB-inoculated silage. A study from Kleinschmit and Kung [25] showed that acetate reduced DM intake. However, Arriola et al. [26] conducted a meta-analysis and found that no effect on DM intake was observed when *Lactobacillus buchneri*-inoculated silages were fed to dairy cows. We attributed the discrepancy in DM intake to the difference in silage fermentation products between inoculant treatment and control. Krizsan et al. [27] found that the reductions in intake were observed in growing cattle with increases in ammonia-N, acetate and propionate in silage. Furthermore, the improvements in intake and performance were mainly due to the higher content of water-soluble carbohydrates (e.g., sugars, fructans) in feeds [28], which further affected the availability of readily fermentable energy for rumen microbiota with potential hydrogenation of fatty acids in rumen [29].

Silage inoculated with a low-temperature-tolerant LAB increased ammonia-N and total VFAs (mainly acetate, propionate and butyrate) and decreased pH in the rumen fluids of yaks. This situation was attributed to the good fermentation of inoculated silages. It is well known that lactate produced by LAB during ensiling is immediately converted into VFA in rumen. Moreover, *Lactobacillus buchneri* in inoculant enhanced the production of acetate and propionate, which directly increased the level of volatile fatty acid in rumen when the inoculated silage was fed to yaks. Propionate was the main substrate for gluconeogenesis in the livers [30], while high acetate in the rumen can reduce the efficiency of energy utilization among ruminants [31]. Thus, the low ratio of acetate/propionate indicated that the rumen fermentation efficiency of silage-contained yak feeds was enhanced by the inoculation of low-temperature-tolerant LAB. Butyrate, which contributed to approximately 70% of the daily metabolic energy of ruminants [32] and butyrate production in rumen played a special role in modulating energy metabolism in the gut ecosystem. Notably, the butyrate content in rumen was higher in LAB-inoculated silage compared with the control. Similar results were from Weinberg et al. [15] who reported that the positive effect of inoculated silages on production of butyrate from lactate was attributed to the dominant ruminal microbes. In addition, the accumulation of butyrate with a high pKa value resulted in a low pH level in the rumen of yaks. A dynamic equilibrium between the synthesis of ammonia-N and the application of microorganisms occurred in the rumen, and the optimum ammonia-N content ranged between 8.5–20 mg/dL [33]. The high ammonia-N in rumen fluid indicated the yaks have abstained adequate nitrogen resources from the increased DM intake. In fact, the inoculated silage provided a higher content of N for the ruminal fermentation of yaks. This suggested that the ruminal fermentation was enhanced when yaks were fed with silages inoculated with a low-temperature-tolerant LAB.

### 3.2. Milk Yield and Basic Chemical Composition of Yaks

As shown in Table 3, inoculated silage significantly increased the yak’s individual milk yield. However, inconsistent results were found by Arriola et al. [26]. Oliveira et al. [13] conducted a meta-analysis and found that the improved milk yield after LAB inoculation was probably attributed to the increased DM intake, which might be caused by reduced accumulation of hyperphagic compounds with a hyperphagic effect in inoculated silages, such as butyrate, ammonia and biogenic amines. Regardless of DM intake, the good preservation (as indicated by the reduced ammonia-N) of protein in LAB-inoculated silage could increase ruminal microbial protein synthesis, and thus contributed to the increase in milk yield [34].

Inoculated silage increased levels of total N, lactose and fat in yak milk compared with control silage. We attributed the positive effect to the inoculation with LAB which improved ruminal function by stimulating activities of the rumen microbiome, increasing VFA production [15], increasing NDF degradability [35] or increasing microbial protein synthesis [34]. In fact, LAB-inoculated silage tended to increase the contents of fat and protein in milk, with no limitation of forage, LAB species, feed type and the level of milk yield of the control cows, but the underlying mechanism was unclear [13]. An assumption was proposed that LAB could not only biohydrogenate and degrade linoleic and linolenic fatty acids during ensiling [36,37], but also reduce the abundance of biohydrogenation intermediates that could potentially inhibit mammary lipogenesis and subsequently milk fat [38]. Furthermore, the inoculation of LAB increased the availability of metabolizable protein and energy for milk protein synthesis by reducing proteolysis and amino acid deamination and decarboxylation during ensiling by enhancing DMI [13].

### 3.3. Amino Acid Composition of Yak Individual Milk

The amino acid composition of yak individual milk was shown in Table 4. With substrates for the growth of starter cultures in process of cheese ripening, the content of free amino acids could affect the technical availability of milk [39]. Lys, Met and His were the first three essential amino acids in yak milk, with a proportion of 2.74–13.15% in total amino acids. Similar results were found by Liu et al. [4]. Previous studies showed that the contents of Lys and Met as limiting amino acids were intensively associated with CP yield of milk [40]. This result was also observed in the current study. Inoculated silage increased (2.68 vs. 1.18 g/100 g, *p* < 0.05) total essential amino acids. This increase was due to elevated contents of Thr (0.21 vs. 0.16 g/100 g, *p* < 0.05), Leu (0.77 vs. 0.61 g/100 g, *p* < 0.05) and Phe (0.35 vs. 0.24 g/100 g, *p* < 0.05). We attributed this trend to the slightly high DM intake, which caused the increased high CP intake in inoculated silages. We also found that inoculated silage increased the ratio of total essential amino acids to non-essential amino acids in yak milk. However, non-essential amino acids in milk showed little change between yaks fed with inoculated silage and control. In fact, some main amino acids, such as Lys, Glu, Arg, Pro, Tyr and Phe, had similar proportions between milk and rumen fluids [41]; the elevation of essential amino acids in feed facilitated the milk yield [42]. Similarly, Liu et al. [4] attributed this result to the protein ratio in forages fed to yaks. Dietary protein in forages firstly provides N for microbial protein synthesis in rumen and then complementing microbial protein to supply amino acids for maintenance, growth, reproduction and milk protein synthesis of yaks. High levels of amino acids were observed in well preserved silage [43]. In the study, therefore, the high quality of inoculated silage partly explained the high content of essential acids in milk.

### 3.4. Fatty Acid Composition of Yak Individual Milk

The fatty acid composition of yak individual milk was shown in Table 5. Palmitic acid (C16:0), oleic acid (sum of trns-11 C18:1, cis-9 C18:1 and cis-11 C18:1) and stearic acid (18:0) were the dominant fatty acids, with a proportion of about 67.5% in the total fatty acids. This value was lower than those (72–74.9%) in yak milk [4]. In addition, inoculated silage increased the content of cis-9 C18:1 in milk. From the perspective of health, unsaturated FA was better than saturated FA, and particular attention was paid to polyunsaturated FA due to its effect in preventing and treating cancer, coronary artery disease, hypertension, diabetes and inflammatory and autoimmune disorders [44]. As expected, LAB-inoculated silage increased the content of polyunsaturated FA, but decreased the content of saturated FA in milk. Recently, Zong et al. [37] also found that inoculation of LAB reduced the loss of polyunsaturated FA during ensiling. Although LAB-inoculated silage increased the content of total long-chain FA, while a decrease in monounsaturated FA and CLA (mainly cis-9 and trans-11 C18:2) and DHA (C22:6) was observed in yak milk. CLA featured anti-carcinogenic, anti-atherogenic, anti-inflammatory and anti-lipogenic effects [45]. We speculated that the poor fermentation with some undesirable microbes in control silage stimulated the increase in CLA in ruminant yak. According to previous reports from Ding et al. [36] and Cui et al. [44], the content of γ-linolenic acid (18:3 n-3) was also detected in yak milk, with no difference between LAB inoculation and control. This fatty acid was helpful to improve vision as well as prevent cancer, cardiovascular diseases and hypertension. Cui et al. [44] proved that levels of linolenic acids and CLA in yak milk depended on the content of fatty acids in feed. Therefore, we attributed the changes in functional fatty acid of yak milk to the improvement of the quality of inoculated silage, even with no data on the fatty acid composition of silages in this study.

## 4. Conclusions

Inoculated silage increased dry matter intake and promoted rumen fermentation, thereby enhancing milk yield and the quality of lactating yaks. In addition, inoculated silage increased concentrations of essential amino acid and polyunsaturated FA in yak individual milk. These confirmed that inoculation of low-temperature-tolerant LAB is an effective and important method to improve the quality of silage and the good performance of lactating yak on the Qinghai–Tibetan Plateau. In the future, studies shall focus on the design of high-quality silage-contained feed for promoting the good performance of lactating yak on the Qinghai–Tibetan Plateau.

## Figures and Tables

**Table 1:** Chemical and microbial compositions of silages fed to yaks.

Item	Control	LAB Inoculation	*p*-Value
DM, %FM	27.59 ± 049	28.18 ± 0.58	0.098
WSC, %DM	2.16 ± 0.21	3.05 ± 0.44	0.043
CP, %DM	6.83 ± 0.31	7.52 ± 0,25	<0.001
NDF, %DM	52.4 ± 1.36	53.1 ± 1.44	0.141
ADF, %DM	31.2 ± 0.94	32.8 ± 0.85	0.165
pH	4.56 ± 0.18	4.18 ± 0.06	0.007
Lactate, %DM	1.63 ± 0.23	2.44 ± 0.34	0.019
Acetate, %DM	0.41 ± 0.21	1.35 ± 0.42	<0.001
Butyrate, %DM	0.13 ± 0.04	0.05 ± 0.02	<0.001
Ammonia-N, %TN	14.6 ± 1.39	9.11 ± 2.44	<0.001
LAB, log10 cfu/g FM	8.97 ± 0.05	9.10 ± 0.04	0.146
Yeasts, log10 cfu/g FM	4.28 ± 0.43	3.35 ± 0.25	0.103

Silages were treated without (control) or with LAB inoculant. ADF, acid detergent fiber expressed inclusive of residual ash; aNDF, neutral detergent fiber assayed with a heat stable amylase and expressed inclusive of residual ash; CP, crude protein; DM, dry matter; FM, fresh matter; FW, fresh weight; WSC, water soluble carbohydrates; LAB, lactic acid bacteria.

**Table 2:** DM intake and yak rumen fermentation products.

Item	Control	LAB Inoculation	*p*-Value
DM intake, kg/d	4.49 ± 0.18	4.71 ± 0.05	0.106
pH	7.24 ± 0.04	7.11 ± 0.05	0.037
Ammonia-N mg/dL	13.51 ± 0.02	24.26 ± 0.02	<0.001
Total VAF, mmol/L	52.93 ± 2.78	60.31 ± 2.44	<0.001
Acetate, mmol/L	38.36 ± 1.52	43.17 ± 1.89	<0.001
Propionate, mmol/L	8.25 ± 1.01	10.29 ± 0.43	<0.001
Butyrate, mmol/L	5.19 ± 0.22	5.73 ± 0.16	<0.001
Isobutyrate, mmol/L	0.32 ± 0.03	0.38 ± 0.01	0.432
Valerate, mmol/L	0.27 ± 0.01	0.30 ± 0.01	0.195
Isovalerate, mmol/L	0.54 ± 0.04	0.44 ± 0.03	0.143
Acetate/Propionate	4.64 ± 0.14	4.19 ± 0.12	0.012

Silages were treated without (control) or with LAB inoculant. DM, dry matter; VFA, volatile fatty acid.

**Table 3:** Milk yield and basic chemical composition of yaks fed with control and inoculated silages.

Item	Control	LAB Inoculation	*p*-Value
Milk yield (kg/d)	1.46 ± 0.11	1.73 ± 0.07	0.021
Total protein (g/100 mL of milk)	4.84 ± 0.11	5.16 ± 0.12	0.027
NPN/TN (%)	4.43 ± 0.06	4.06 ± 0.05	<0.01
WPN/TN (%)	23.06 ± 0.93	24.11 ± 0.82	0.169
α-lactalbumin	0.04 ± 0.01	0.05 ± 0.01	0.379
β-lactoglobulin	0.58 ± 0.08	0.66 ± 0.05	0.065
CN/TN (%)	72.51 ± 1.19	71.83 ± 0.93	0.080
WPN/CN (%)	31.80 ± 1.51	33.57 ± 1.49	0.051
Fat (g/100 mL of milk)	7.45 ± 0.27	8.05 ± 0.21	0.032
Saturated FA	69.34 ± 0.49	70.67 ± 0.53	<0.001
Monounsaturated FA	62.79 ± 1.21	59.63 ± 1.02	<0.001
Polyunsaturated FA	28.9 ± 3.11	35.82 ± 2.08	<0.001
Short-chain FA	11.25 ± 0.42	12.83 ± 0.81	0.321
Medium-chain FA	35.74 ± 0.53	34.47 ± 0.39	0.114
Long-chain FA	47.96 ± 1.44	50.98 ± 0.75	<0.001
Lactose (g/100 mL of milk)	4.91 ± 0.25	5.46 ± 0.12	0.010
Total soilds (g/100 mL of milk)	15.31 ± 0.39	16.89 ± 0.73	0.094

CN, casein nitrogen; FA, fatty acid; NPN, non-protein nitrogen; WPN, whole protein nitrogen.

**Table 4:** Amino acid composition (g/100 g milk) of yak individual milk.

Item	Control	LAB Inoculation	*p*-Value
Essential Amino-Acid (EAA)			
Thr	0.16 ± 0.03	0.21 ± 0.01	<0.001
Val	0.27 ± 0.02	0.33 ± 0.02	0.061
Met	0.08 ± 0.01	0.10 ± 0.01	0.084
Ile	0.19 ± 0.03	0.25 ± 0.02	0.072
Leu	0.61 ± 0.02	0.77 ± 0.04	<0.001
Phe	0.24 ± 0.02	0.35 ± 0.02	<0.001
Lys	0.48 ± 0.02	0.51 ± 0.03	0.106
His	0.15 ± 0.01	0.16 ± 0.01	0.129
Trp	<0.01	<0.01	0.087
Total EAA (TEAA)	2.18 ± 0.13	2.68 ± 0.22	<0.001
Non-essential amino acid (NEAA)			
Cys	0.02 ± 0.01	0.03 ± 0.01	0.126
Arg	0.14 ± 0.01	0.15 ± 0.01	0.091
Pro	0.51 ± 0.02	0.46 ± 0.03	0.054
Asp	0.27 ± 0.01	0.29 ± 0.02	0.077
Ser	0.32 ± 0.04	0.25 ± 0.05	0.152
Glu	1.27 ± 0.09	1.51 ± 0.03	0.033
Gly	0.18 ± 0.02	0.22 ± 0.02	0.065
Ala	0.10 ± 0.01	0.11 ± 0.01	0.179
Tyr	0.2 ± 0.03	0.15 ± 0.02	0.145
Total NEAA (TNEAA)	3.01 ± 0.07	3.17 ± 0.09	0.059
Total ammo acid (TAA)	5.19 ± 0.16	5.85 ± 0.23	<0.001
TEAA/TAA	0.42 ± 0.02	0.46 ± 0.03	0.073
TNEAA/TAA	0.58 ± 0.03	0.54 ± 0.03	0.085
TEAA/TNEAA	0.72 ± 0.05	0.85 ± 0.04	0.018

Yaks were fed with control and inoculated silages.

**Table 5:** Fatty acid composition (g/100 g fatty acid) of yak individual milk.

Item	Control	LAB Inoculation	*p*-Value
C4:0	4.33 ± 0.10	4.65 ± 0.13	0.078
C6:0	3.58 ± 0.23	3.92 ± 0.21	0.143
C8:0	1.08 ± 0.18	1.31 ± 0.12	0.053
C10:0	2.01 ± 0.22	2.64 ± 0.23	0.002
C11:0	0.25 ± 0.04	0.31 ± 0.06	0.065
C12:0	1.49 ± 0.16	1.20 ± 0.15	0.051
C13:0	0.05 ± 0.05	0.18 ± 0.04	0.022
C14:0	7.06 ± 0.07	5.44 ± 0.08	<0.001
C14:1	0.42 ± 0.04	0.38 ± 0.02	0.094
C15:0	0.87 ± 0.26	1.23 ± 0.29	0.139
C15:1	1.25 ± 0.33	1.64 ± 0.27	0.103
C16:0	23.29 ± 0.68	22.42 ± 0.70	0.261
C16:1	1.31 ± 0.14	1.98 ± 0.13	<0.001
C17:0	0.45 ± 0.25	0.76 ± 0.18	0.066
C17:1	0.88 ± 0.38	1.43 ± 0.52	0.073
C18:0	16.73 ± 2.14	14.17 ± 2.43	0.119
Trans-11 C18:1	3.03 ± 0.08	4.15 ± 0.11	<0.001
cis-9 C18:1	20.72 ± 0.91	24.54 ± 0.79	<0.001
cis-11 C18:1	0.79 ± 0.22	1.32 ± 0.15	<0.001
18:2 n-6	1.48 ± 0.03	1.51 ± 0.02	0.069
C18:3 n-3	0.13 ± 0.01	0.15 ± 0.01	0.054
cis-9,trans-11 CLA	1.12 ± 0.12	0.74 ± 0.15	<0.001
C20:0	0.43 ± 0.06	0.31 ± 0.09	0.092
C20:1 n-9	0.59 ± 0.08	0.44 ± 0.03	0.037
C20:2	0.02 ± 0.01	0.01 ± 0.01	0.054
C21:0	0.05 ± 0.01	0.04 ± 0.01	0.088
C20:3 n-6	0.09 ± 0.01	0.08 ± 0.01	0.081
C20:4	0.17 ± 0.03	0.11 ± 0.03	0.105
C20:3 n-3	0.06 ± 0.01	0.05 ± 0.01	0.143
C22:0	0.47 ± 0.09	0.31 ± 0.07	0.051
C20:5 EPA	0.07 ± 0.01	0.04 ± 0.01	<0.001
C22:1	0.01 ± 0.00	0.01 ± 0.00	0.273
C23:0	0.21 ± 0.02	0.14 ± 0.01	<0.001
C24:0	0.23 ± 0.07	0.46 ± 0.04	<0.001
C24:1	0.15 ± 0.03	0.09 ± 0.01	<0.001
C22:6 DHA	0.08 ± 0.02	0.12 ± 0.01	0.041

Yaks were fed with control and inoculated silages. CLA, conjugated linoleic acid; DHA, docosahexaenoic acid; EPA, eicosapentaenoic acid; FA, fat acid; Long-chain FA, sum of C17:0 to C24:0; Medium-chain FA, sum of C12:0 to C16:1; Saturated FA, sum of C4:0 to C24:0; Short-chain FA, sum C4:0 to C11:0.

## Data Availability

All data are presented in the text and tables of this manuscript.
